# Investigation of the playing digital games on shoulder flexibility, muscle strength and reaction speed in volleyball players

**DOI:** 10.3389/fpubh.2024.1493900

**Published:** 2024-10-30

**Authors:** Şeyma Güney, Fatma Neşe Şahin, Cansel Arslanoğlu, Özkan Güler, Mert Aydoğmuş, Abdullah Doğan, Tebessüm Ayyıldız Durhan, Gökhan Arıkan, Onur Mutlu Yaşar, Hamza Küçük

**Affiliations:** ^1^Department of Health and Sport Sciences, Institute of Health Sciences, Physical Activity, Ankara University, Ankara, Türkiye; ^2^Department of Coaching Education, Faculty of Sport Sciences, Ankara University, Ankara, Türkiye; ^3^Department of Coaching Education, Faculty of Sport Sciences, Sinop University, Sinop, Türkiye; ^4^Department of Coaching Education, Faculty of Sport Sciences, Ankara University, Ankara, Türkiye; ^5^Department of Coaching Education, Hasan Dogan Faculty of Sport Sciences, Karabuk University, Karabuk, Türkiye; ^6^Ministry of Youth and Sports, Ankara, Türkiye; ^7^Department of Recation, Faculty of Sport Sciences, Gazi Univesity, Ankara, Türkiye; ^8^Mehmet Arabaci School of Physical Education and Sport, Harran University, Sanliurfa, Türkiye; ^9^Faculty of Health Sciences, Izmir Democracy University, Izmir, Türkiye; ^10^Yasar Dogu Faculty of Sport Sciences, Ondokuz Mayis University, Samsun, Türkiye

**Keywords:** physical performance, e-sports, shoulder flexibility, muscle strength, physical exercise, volleyball players

## Abstract

Esports is a natural extension of digital games. Digital games provide a platform for players to showcase their skills, thereby forming the foundation of e-sports. These two domains continuously support each other, demonstrating significant growth in popularity and establishing a solid presence in the competitive landscape. Digital games require high levels of attention, strategy, reflexes, and coordination, offering pathways to reach the pinnacle of competition. The benefits derived from digital games enhance the ability to perform effectively under stress, making them crucial for both physical and mental performance. This study investigates the effects of digital gaming on the physical performance, shoulder flexibility, muscle strength and reaction speed of female volleyball players. A total of 50 female volleyball players took part, who were divided into two groups based on their digital playing habits: Group 1 consisted of female players who played video games for at least 4 h per day in the last 6 months, while Group 2 included those who played <4 h per day. In the study, the Activ5© Handheld Digital Dynamometer was used to measure the strength of the shoulder muscles during various movements, including flexion, hyperextension, abduction, internal rotation and external rotation. In addition, the BlazePod™ trainer device was used to assess reaction times. The results showed that volleyball players who frequently played digitally showed significant improvements in shoulder flexibility, especially in flexion of the non-dominant side. In addition, these players showed faster reaction times than those who played less frequently. However, a significant decrease in hyperextension muscle strength was observed in the group that frequently played digital games. The study highlights that while digital games can improve certain physical skills such as reaction speed and shoulder flexibility, they can also contribute to muscular imbalances or a decrease in muscle strength in certain areas. These findings suggest potential benefits for volleyball players, particularly in movements that require quick reflexes and flexibility, such as serving and spiking. However, the results also raise concerns about potential negative effects on muscle strength and posture, highlighting the need for balanced training programs to mitigate these effects. Further research is needed to investigate the long-term effects of digital games on athletic performance and physical health.

## 1 Introduction

Volleyball is a sport that requires high and low intensity activities, technical and tactical skills, and versatile performance such as high endurance, strength, speed, flexibility, and balance (1). While the actions performed by the players may differ in terms of the technical and tactical requirements of their individual roles, common movements include acceleration and deceleration, jumping, kicking, and multi-directional movement (2, 3). To perform well, players must have advanced physical and physiological capacities and important motor control and cognitive functions (4–7). In volleyball, especially during the attack, coordinative abilities such as quick strength and continuity in strength are important. Technological advancements are effectively utilized to enhance technical skills and physical performance in volleyball players. These developments contribute to the assessment and improvement of players' techniques, extending beyond applications such as match analysis, data monitoring, and match analysis. Furthermore, these technological innovations provide performance enhancements in areas that require attention, offering new trends aimed at the development of athletes.

Technology has affected sports and provided the emergence of new sports branches. One of these sports branches is e-sports (8). E-sports is any activity that is participated in offline or online, as a team or individually, via electronic device (9). E-sports, like volleyball, requires quick movements to control the game. One of the determinants of speed is muscle strength and muscle strength can increase in direct proportion to the performance of the video game (10). Playing games on the console for a long time will increase shoulder-muscle activity; It has been scientifically proven that sitting in the anterior position of the head for 5 minutes causes a significant reduction in shoulder external rotator muscle strength (11, 12). Considering that the competitions last for hours, the importance of anaerobic endurance in e-sports players emerges. One of the important factors in e-sports is the ability to react quickly (13). Neurocognitive research has shown that video game gamers display some superior cognitive characteristics (14). Video games require speed and concentration and these skills significantly reduce correct reaction times (15). Developing cognitive abilities in this way is vital to the success of the volleyball player; good performance is directly related to their capacity to anticipate and respond to changes in the environment (16). The ability to anticipate and adapt, crucial for cognitive development, can be further enhanced by practical applications targeting key physical attributes such as reaction time, strength, and endurance.

The existence of an application that affects reaction time, strength, and endurance performance in volleyball athletes has a great importance in the quality of sports skills (15). E-sports; It can contribute to the development of reaction time, strength and endurance in volleyball athletes.

Previous studies have mostly focused on negative effects on health-related component of physical fitness. The common sense about e-sports is, due to fact that players perform limited physical activities, it is a sedentary behavior. These actions may cause negative changes on the physical fitness parameters of e-sports players. This study is important in terms of investigating the effect of e-sports on selective performance-related component of physical fitness. On the contrary to previous studies, and limited national and international literature, present investigation may find out positive effects of e-sport on athlete selective performance. Parameters like shoulder flexibility, strength, and reaction time.

Important training models play a role in the development of physical performance. These training interventions aim to systematically and sustainably improve performance. Coordination/skills, condition, and cognitive-tactical skills are among these “building blocks” (17). There are different training interventions for performance development in athletes, and when repeated, these interventions can lead to monotony. Therefore, the impact of various interventions is as essential as training models. Digital games can serve as a valuable complementary resource that facilitates the multidimensional development of athletes, contributing to the holistic development of both physical and mental performance. This study aims to examine the effects of digital game playing status of volleyball players on shoulder flexibility, muscle strength, and reaction time.

## 2 Material and methods

### 2.1 Research group

The 1^st^ and 2^nd^ league female volleyball players of Ilbank Sports Club and Karayollari Sports Club voluntarily participated in the research. Participants were divided into 2 groups: For the 1^st^ group; 25 volleyball athletes who played video games for a minimum of 4 h per day for at least the past 6 months. For the 2^nd^ group; 25 volleyball athletes who have played video games < 4 h a day for at least the past 6 months.

The research group was determined through a subject information form. The form was filled by 54 volunteers. According to the results of the subject information form, 50 volleyball player who met the inclusion criteria were included and 4 volleyball player was excluded. Demographic data of participants is shown in Table 1.

**Table 1:** Demographic data of participants.

	**1. Group (*****n*** **=** **25)**		**2. Group (*****n*** **=** **25)**	
	**Mean**	**SD**	**Mean**	**SD**
Age	17.36	3.30	20.48	4.57
Height (cm)	177.92	5.09	181.00	6.87
Weight (kg)	63.98	6.25	67.76	7.51

### 2.2 Testing shoulder flexibility with digital goniometer

In the study, flexion, hyperextension, abduction, internal rotation, and external rotation shoulder joint range of motion were evaluated. A digital goniometer was used as a measurement tool. The Activ5© uses a Bluetooth-enabled sensor to measure the range of motion displayed to the user via a smartphone app. It enables a wireless connection to a mobile phone, providing the convenience of displaying the test score remotely. A study evaluating the reliability and concurrent validity of active shoulder flexion, abduction, external, and internal rotation, digital goniometer measurements equivalent to the Activ5© digital hand dynamometer demonstrated reliability coefficients (recommended threshold for making clinical decisions) exceeding 0.90 (18). After the people were given the correct position for measurement, the movements were explained in detail and in practice. Measurements were made bilaterally and were repeated 3 times for a healthy measurement.

### 2.3 Testing shoulder muscle strength with digital dynamometer

Shoulder flexion muscle strength (MS), hyperextension muscle strength, abduction muscle strength, external rotation muscle strength, and internal rotation muscle strength were evaluated using the Activ5© Handheld digital dynamometer in the study. The Activ5© is a HHD which can measure isometric strength (N or kg) and uploads the data immediately to a smartphone app. The Activ5 showed excellent results in a test- retest using the Instron ElectroPuls mechanical tester (a gold standard system) and was considered reliable (with ICC ≥ 0.971 for test-retest reliability) (19).

Volleyball athletes resisted the applied force in the tested position. The value seen on the digital hand dynamometer was recorded as Newton (N). Measurements were applied to both the dominant arm and non-dominant arm.

### 2.4 Testing reaction times with visual-cognitive technology—BlazePod™

The reaction times of the volleyball athletes in the hand-eye coordination test were carried out with the BlazePod™ Trainer Device (Play Coyotta Ltd., Tel Aviv, Israel), which consists of wireless light six discs that can be controlled with an application compatible with smart devices. The reliability and validity of the BlazePod system has been established in past studies (20, 21). Athletes were instructed to turn off the light signal as quickly as possible by touching the light sensor with their dominant hand when the light signal was turned on. Light discs were placed on a plate/floor at 20 cm intervals from the designated starting point and 45 cm from the center. Before starting the test, the athletes were allowed to take a pre-test. For each participant, the measurement began with the researcher manually tapping the “start now” button in the BlazePod phone app. After the “3-2-1-continue” warning sound stopped after the start command, the sensors started to flash randomly for 30 seconds. In this study, taking the measurement method of De-Oliveira et al. (20) as an example, the athletes were contacted for 30 seconds to determine their reaction times and 1-min rest was given between sets. Participants repeated this action until the end of the test period. The test was repeated three times. The best value obtained was recorded. The total number of hits and the mean reaction time were recorded.

### 2.5 Statistical analysis

IBM SPSS 24.0 package program (SPSS Inc., Chicago, IL, USA) was used in the statistical analysis of the data obtained from the study. The normality distribution of the data was carried out by Shapiro Wilk analysis since the number of participants was < 50 in both groups. In the comparison of the within-group means, the Paired Sample *t-*Test, which is a parametric test, was used for the mean differences in the data where the mean differences were normal. Independent *t*-Test was applied for statistical analysis of means between groups. Groups are expressed as means ± standard error. The significance value was accepted as 0.05 in all statistical analyzes.

## 3 Results

In the flexibility evaluations, the non-dominant shoulder flexion flexibility of the group playing digital games was found to be significantly higher than the group that do not play digital games (t = 2.092; *P* = 0.042) (Table 2).

**Table 2:** Comparison of shoulder flexibility values between groups.

		**1. Group Mean ±SD**	**2. Group Mean ±SD**	**t**	***p***
Flexion flexibility	Dominant	192.065 ± 11.48	188.03 ± 10.89	1.273	0.209
	Non-dominant	194.7 ± 9.84	188.8 ± 10.09	2.092	**0.042**
Hyperextension flexibility	Dominant	92.25 ± 15.67	85.67 ± 14.06	1.562	0.125
	Non-dominant	90.02 ± 16.54	85.08 ± 14.22	1.132	0.263
Abduction flexibility	Dominant	184.72 ± 19.84	186.72 ± 12.6	−0.425	0.672
	Non-dominant	187.13 ± 27.01	186.42 ± 10.35	0.123	0.903
Internal rotation flexibility	Dominant	66.62 ± 13.42	66.43 ± 12.67	0.051	0.960
	Non-dominant	72.04 ± 14.9	71.7 ± 9.24	0.070	9.450
External rotation flexibility	Dominant	111.67 ± 11.23	111.42 ± 11.64	0.077	9.390
	Non-dominant	111.73 ± 11.49	110.01 ± 10.63	0.546	0.586

In the muscle strength evaluations, the hyperextension muscle strength on the non-dominant shoulder of the group that playing digital games was found to be significantly lower than the group that do not playing digital games (t = −3.284; *p* = 0.002) (Table 3).

**Table 3:** Comparison of shoulder strength values between groups.

	**1. Group Mean ±SD**	**2. Group Mean ±SD**	**t**		***p***
Flexion MS	Dominant	99.94 ± 19.53	97.01 ± 9.63	0.673	0.504
	Non Dominant	90.75 ± 19.33	91.85 ± 12.82	−0.235	0.815
Hyperextension MS	Dominant	80.34 ± 12.13	84.64 ± 7.19	−1.522	0.134
	Non Dominant	68.25 ± 11.52	78.62 ± 10.79	−3.284	**0.002**
Abduction MS	Dominant	89.44 ± 16.70	88.19 ± 13.01	0.295	0.770
	Non Dominant	81.15 ± 17.78	83.53 ± 13.04	−0.540	0.591
Internal Rotation MS	Dominant	146.42 ± 32.76	150.74 ± 18.62	−0.573	0.569
	Non Dominant	138.47 ± 27.65	141.02 ± 15.87	−0.401	0.690
External Rotation MS	Dominant	117.14 ± 16.26	126.81 ± 19.15	−1.924	0.500
	Non Dominant	115.12 ± 17.57	120.94 ± 18.93	−1.128	0.265

In the shoulder muscle strength evaluations of the group that playing digital games, Flexion (*p* = 0.002), Hyperextension (*p* = 0.001), Abduction (*p* = 0.001), and Internal Rotation (*p* = 0.031) muscle strengths on the dominant shoulder were found to be significantly higher than on the non-dominant shoulder (Table 4).

**Table 4:** Comparison of in-group shoulder muscle strength values of the group playing digital games.

	**Dominant**		**Non-dominant**		***p***
	**Mean**	**SD**	**Mean**	**SD**	
Flexion MS	99.94	19.53	90.75	19.33	**0.002**
Hyperextension MS	80.34	12.12	68.25	11.52	**0.001**
Abduction MS	89.44	16.7	81.15	17.78	**0.001**
Internal rotation MS	146.42	32.76	139.47	27.65	**0.031**
External rotation MS	117.14	16.26	116.12	17.57	0.518

In the shoulder muscle strength evaluations of the group that do not playing digital games, Flexion (*p* = 0.014), Hyperextension (*p* = 0.022), Abduction (*p* = 0.044), Internal Rotation (*p* = 0.007), and External Rotation (*p* = 0.033) muscle strength on the dominant side were found to be significantly higher than on the non-dominant side (Table 5).

**Table 5:** Comparison of in-group shoulder muscle strength values of the group do not playing digital games.

	**Dominant**		**Non-dominant**		***p***
	**Mean**	**SD**	**Mean**	**SD**	
Flexion MS	97.01	9.63	91.85	12.82	**0.014**
Hyperextension MS	84.64	7.19	78.67	10.79	**0.022**
Abduction MS	88.19	13.01	83.53	13.04	**0.044**
Internal rotation MS	150.74	18.62	141.02	15.87	**0.007**
External rotation MS	126.81	19.15	120.94	18.93	**0.033**

In the reaction time evaluations, the reaction time of the group playing digital games was found to be significantly lower on the dominant side (t = −2.135; *p* = 0.038) and on the non-dominant side (t = −2.460; *p* = 0.018) compared to the group do not playing digital games. The reaction hits on the non-dominant side of the group playing digital games were found to be significantly higher than the group that do not playing digital games (t = 2.245; *p* = 0.029), but the reaction hits on the dominant side did not differ significantly (*p* > 0.05) (Table 6).

**Table 6:** Comparison of reaction times values between groups.

		**1. Group Mean ±SD**	**2. Group Mean ±SD**	**t**	***p***
Reaction time	Dominant	421.36 ± 30.40	440.08 ± 31.57	−2.135	**0.038**
	Non-Dominant	434.60 ± 33.60	457.00 ± 30.71	−2.46	**0.018**
Reaction hits	Dominant	66.12 ± 4.11	63.36 ± 4.13	2.366	0.220
	Non-Dominant	64.28 ± 4.26	61.68 ± 3.91	2.245	**0.029**

In the reaction time evaluations of the digital game playing group, the reaction time on the dominant side was found to be significantly lower than on the non-dominant side (*p* = 0.001). In the digital game playing group, Reaction Hits on the dominant side were found to be significantly higher than on the non-dominant side (*p* = 0.001) (Table 7).

**Table 7:** Comparison of in-group reaction times values of the group playing digital games.

	**Dominant**		**Non-dominant**		***p***
	**Mean**	**SD**	**Mean**	**SD**	
Reaction time (ms)	421.36	30.4	434.6	33.6	0.001
Reaction hits	66.12	4.11	64.28	4.26	0.001

In the evaluations of the reaction time of the group who do not play digital games, the reaction time on the dominant shoulder was significantly lower than on the non-dominant shoulder (*p* = 0.002). In the group that do not play digital games, Reaction Hits on the dominant side were found to be significantly higher than on the non-dominant side (*p* = 0.004) (Table 8).

**Table 8:** Comparison of in-group reaction times values of the group do not playing digital games.

	**Dominant**		**Non-dominant**		
	**Mean**	**SD**	**Mean**	**SD**	***p***
Reaction time (ms)	440.08	31.57	457.00	30.71	0.002
Reaction hits	63.36	4.13	61.68	3.91	0.004

## 4 Discussion

The aim of our study was to examine the effects of playing digital games on shoulder flexibility, shoulder muscle strength and reaction speed of female volleyball players. Flexibility evaluation results revealed that the non-dominant side flexion flexibility of the group that playing digital games increased significantly compared to the group that do not playing digital games. However, playing digital games; Compared to those who do not play digital games, no significant effect was found on the flexibility of the dominant shoulder Flexion, Hyperextansion, Abduction, Internal Rotation, External rotation on both the dominant, and non-dominant side shoulder flexibility (Table 2). Digital gaming was found to significantly reduce hyperextension muscle strength on the non-dominant shoulder compared to the non-digital gaming group. Playing digital games did not make a significant difference in the hyperextension muscle strength on the dominant side, flexion, abduction, internal rotation, and external rotation in both dominant and non-dominant shoulder muscle strength compared to the group that do not play digital games (Table 3). In the reaction time evaluations, it was found that the reaction time of the group playing digital games improved significantly on the dominant shoulder and on the non-dominant shoulder compared to the group do not playing digital games. Reaction hits on the non-dominant side were found to be significantly higher in the group that played digital games compared to the group that do not, but reaction hits on the dominant side did not differ significantly (Table 6).

This research revealed that shoulder flexibility, except flexion flexibility, is not affected by digital game playing (Table 2). The predominantly seated nature of digital gaming may contribute to this issue. Individuals engaged in digital gaming can remain in static positions for extended periods in poor postures, significantly impact shoulder flexibility (22). Similar to our study, Straker et al. (23), in a study investigating the posture and muscle activities of young children during the use of tablet computers, desktop computers and paper; found that shoulder flexion increased significantly especially during tablet use. Bullock et al. (24), in his study investigating the effects of sitting position on shoulder pain and range of motion in 28 subjects with shoulder impingement; significantly greater flexion ROM was recorded in an erect sitting posture compared to in a slouched posture.

Another reason for the decrease in shoulder flexibility may be microtraumas caused by repetitive actions. Hakala et al. (25) observed that excessive engagement in gaming and computer usage are associated with increased risk of experiencing body pain, while TV viewing does not exhibit the same correlation. Players need to rapidly move their upper extremities when playing games, and these physical burdens cause muscle stiffness in the shoulder girdle (26). The overload can lead to stiffness in the trapezius muscle, causing the scapula to become displaced (27). The scapular position is believed to influence overall shoulder girdle function because there are many muscle connections between the spine, humerus, scapula, and collarbone (28). Kebaetse et al. (28) investigated the effects of thoracic posture on scapular movement patterns, shoulder range of motion and strength in 34 asymptomatic subjects. Significantly less active shoulder abduction ROM was noted in a slouched posture. Contrary to this study, no significant change was observed in shoulder abduction flexibility in our study (Table 2). However, in our study, individuals maintain a certain sitting posture by playing digital games for at least 4 h a day for at least 6 months. This may result in persistent changes in the shoulder flexibility.

In our study, it was determined that playing digital games significantly reduced shoulder hyperextension muscle strength on the non-dominant side (Table 3). In addition, when all evaluated muscle strengths are compared, all muscle strengths in all directions except external rotation are high in the dominant shoulder for both groups (Tables 4, 5). However, there was no significant different in the dominant shoulder in the group playing digital games (Table 4). The decrease in external muscle strength in the dominant shoulder of the group playing digital games may suggest the effect of digital game on reducing muscle strength. Puolitaival et al. (29) compared adolescent men who played video games for more than 3 h a day on weekdays, similar to our study method, with adolescent men who played 3 h or less per day on weekdays. As a result of the research, it was found that the grip strength of the participants who played video games for more than 3 h a day on weekdays was 2.1% less. This may be due to abnormal repetitive movements or constant static postures while playing digital games (26). Maintaining postures while playing digital games causes several muscles to be in elongated positions while other muscles are placed in shortened positions. This change in length between the muscles is assumed to cause muscle weakness that causes muscle imbalance (30). One of the most common problems that occur as a result of these certain postures when playing digital games is forward head posture. Examining the prevalence problem of forward head posture in electronic players and related factors among electronic players, Ashok et al. (31) found that there was no relationship between the gaming device and forward head posture, and that the risk of developing forward head posture in gamers was significantly high. Villanueva et al. (32), in their study examining the relationship between sitting stance and shoulder and neck muscle activities, found increased muscle load in shoulder and neck electromyographic (EMG) activities of forward-leaning posture by changing screen height. Even a minor deviation from a neutral shoulder position has been associated with increased shoulder muscle load (32). Less active muscles have less load, more active muscles have more load (33). A systematic review found that muscle activity in the shoulder and forearm region increases as repeated upper extremity movements activate a sustained muscle contraction while using a smartphone (34). Niemi et al. (35) found that computer use required static loading of the upper extremities and was positively associated with shoulder pain in women. There are studies in which sitting posture significantly affects shoulder muscle strength (28, 36).

Video games require speed and concentration and these skills significantly reduce correct reaction times (15). People typically do 30 to 300 actions per minute (APM), which increases with player skill, with professional players often going over 500 APM (37). In terms of athletes, reaction times play a key role in achieving high performance and preventing injuries (38). Therefore, the improvement in reaction time can positively affect the performance of the volleyball player. There are many studies investigating the effect of playing digital games on reaction time. Lager and Bremberg (39) reported that digital games have supportive effects on reaction time and spatial perception in 30 studies they examined. The study noted improvement in reaction times for players who played video games for 14 to 33 h over a few months. Yuji (40) stated that video game players had faster reaction times to stimuli on discrimination perception tests using computers compared to non-players. In a study conducted by Goldstein et al. (41), older adults were randomly assigned to either an experimental group, where they played video games for 5 or more hours per week for a duration of 5 weeks, or a control group that did not engage in video gaming. The results of pre-experimental and post-experimental reaction time assessments revealed that participants in the experimental group exhibited significantly faster reaction times compared to those in the control group. The results of our study found that digital game improves reaction time and supports the literature. While the reaction hits on the non-dominant side of the playing digital game group were found to be significantly higher than the do not playing digital game group; The reaction time was significantly lower in both the dominant shoulder and non-dominant shoulder of the digital gaming group (Table 6).

## 5 Conclusions

In this study, the effects of playing digital games on shoulder flexibility, shoulder muscle strength and reaction time in female volleyball players were investigated. It was found that playing digital games significantly increased shoulder flexion on the non-dominant side. For muscle strength; It was observed, among groups, that playing digital games significantly reduced hyperextension muscle strength on the non-dominant shoulder. It is also thought that digital game affects external muscle strength on the dominant side. However, no significant effect of digital game on shoulder flexion, abduction, and internal rotation muscle strength was observed. Finally, it has been shown that playing digital games provides improvements by reducing reaction time.

Athletes who have sports branches where upper extremity flexibility, muscle strength and reaction time are important can benefit from the results of this research. Because each individual has a unique genetic, the results may not be the same for every athlete. Digital games can improve volleyball players' performances that require flexibility, such as spike and serve, which affect success. It can increase sports performance, especially by improving reaction speed. The length-tension relationship maintained during the game can increase the success of sports by providing muscle development. On the other hand, the lack of physical activity required by the digital game and the loss of muscle strength caused by the digital game can be compensated by the training program of the athlete. At the same time, this study may serve as a warning to digital game players, as the fixed posture maintained during the game may cause musculoskeletal problems.

The fact that the study was conducted exclusively with female volleyball players can be considered a limitation of the research. Future studies may investigate the effects of digital games on physical performance in both male and female volleyball players. Another limitation is the lack of clarity regarding the type of gaming device used and the specific digital games played. It is recommended that new research examine different types of digital games and various e-sports games. Additionally, assessing the effects of digital games on different physical performance parameters is crucial.

## Data Availability

The raw data supporting the conclusions of this article will be made available by the authors, without undue reservation.
